# Psychological Resources Are Independently Associated with Markers of Inflammation in a Middle-Aged Community Sample

**DOI:** 10.1007/s12529-016-9553-z

**Published:** 2016-03-15

**Authors:** Ina Marteinsdottir, Jan Ernerudh, Lena Jonasson, Margareta Kristenson, Peter Garvin

**Affiliations:** 1 Division of Neuro and Inflammation Sciences, Department of Clinical and Experimental Medicine, Linköping University, 581 83 Linköping, Sweden; 2 Department of Medical and Health Sciences, Division of Cardiovascular Medicine, Linköping University, Linköping, Sweden; 3 Division of Community Medicine, Department of Medical and Health Sciences, Linköping University, Linköping, Sweden; 4 Unit for Research and Development of Local Health Care, County of Östergötland Linköping, Sweden

**Keywords:** Inflammation, Biomarker, Positive psychology, self-anchoring ladder, Psychoneuroimmunology, Epidemiology

## Abstract

**Purpose:**

To elucidate possible independent associations of psychological resources with inflammatory markers, all linked with coronary heart disease (CHD).

**Method:**

In a middle-aged general population (*n* = 944), psychological resources (coping, self-esteem, and sense of coherence (SOC)), a global measure of quality of life (Cantril’s self-anchoring ladder, also called “ladder of life”), and psychological risk factors (hopelessness, vital exhaustion, and depressive symptoms) were used in linear regression models to evaluate associations with the inflammatory markers interleukin (IL)-6, C-reactive protein (CRP), and matrix metalloproteinase (MMP)-9. Adjustments were done for age, sex, medical conditions, and cardiovascular risk factors.

**Results:**

After full adjustments, self-esteem was independently associated with all three biomarkers. Ladder of life was associated with IL-6 and log-CRP; coping, vital exhaustion, and depressive symptoms with IL-6; and SOC with MMP-9 (*p* < 0.05 for all associations).

**Conclusion:**

Numerous significant associations of psychological resources and risk factors with IL-6, CRP, and MMP-9 were found in a community-based sample. The associations of psychological resources were mostly independent, while the psychological risk factors seemed preferentially dependent on lifestyle factors as smoking, physical activity, and body mass index (BMI). This suggests that the psychological resources’ (in particular self-esteem) protective effects on CHD are linked to inflammatory markers.

## Introduction

There is abundant evidence that psychological distress is independently associated with risk for somatic disease and mortality. More specifically, perceived stress [1, 2], anxiety [2], depressiveness [2–5], vital exhaustion [6], and hopelessness [7] have been prospectively associated with incidence and/or mortality of coronary heart disease (CHD). Recently, a discussion on positive psychology has emerged, raising the question whether there are shared or unique contributions to health outcomes when comparing psychological risk factors to psychological resources. As for CHD, there are so far only a few prospective analyses on the protective effect of psychological resources. It has been shown that mastery (coping) [8], sense of coherence (SOC) [8], optimism [9], emotional vitality [10], and perceived life enjoyment [11] are associated with a lower incidence of CHD.

Recently, we confirmed the findings on mastery and SOC and added the knowledge that also self-esteem was independently associated with lower risk of first-time CHD events, in an 8-year follow-up of a middle-aged community-based population [12]. In particular, it was concluded that self-esteem seems to be of high relevance, as its cardioprotective effect remained after adjustment for depression or hopelessness [12]. This raises the question through which pathways psychological factors exert their effects of CHD.

As CHD is regarded as an inflammatory disease and inflammatory markers such as C-reactive protein (CRP) and interleukin (IL)-6 are well known predictors of incident CHD [13–15], it has been postulated that there is an association between inflammatory markers and psychological factors. In the rapidly growing field of psychoneuroimmunology [16, 17], associations between psychological risk factors and inflammatory biomarkers have been frequently studied, with IL-6 and CRP as the most studied biomarkers. Associations have been reported between IL-6 or CRP and depression [18–20], hopelessness [21], vital exhaustion [20, 21], negative affect, and psychological distress [22]. In addition, we have previously shown that psychological risk factors are associated with matrix metalloproteinase (MMP)-9 [23], a collagen-degrading enzyme that is up-regulated in inflammation and involved in the development of atherosclerotic plaques [24]. Like CRP and IL-6, MMP-9 has been shown to predict new CHD events [25].

Importantly, in contrast to risk factors, there are few reports of associations of psychological resources with inflammatory markers in normal population samples [26]. A potential association has been suggested, but findings from large-scale population-based studies are few and mixed [26]. Therefore, more studies on the relation between inflammation and psychological resources are warranted. Such studies could elucidate whether the association between psychological resources and inflammatory markers are confounded by other cardiovascular risk factors or if the association is independent. We have previously reported independent associations of the psychological resources coping, self-esteem, and SOC, with IL-6 in a selected sample from a normal population [21], and also of coping, self-esteem, and SOC with MMP-9 in a subset of the present sample [23].

The aim of this study was to investigate whether psychological resources with reported CHD protective effects are associated, either independently or not from cardiovascular risk factors, with three functionally different inflammatory markers related to CHD incidents, namely, IL-6, CRP, and MMP-9. Our hypothesis was that psychological resources would be associated with inflammatory markers, independent of cardiovascular risk factors.

## Methods

### Study Population and Procedures

The sample constitutes the baseline of the prospective Life Conditions, Stress, and Health (LSH) study [27], with the main aim to investigate to what extent psychosocial factors and biological markers for stress and inflammation can explain differences of incident myocardial infarction in relation to socioeconomic status.

Data collection was performed in collaboration with ten primary healthcare centers (PHCs) in the county of Östergötland in the southeast of Sweden. All citizens in the age range of 45 to 69 years living in any of the catchment areas of the ten PHCs were eligible for invitation.

The participants were invited consecutively to reach a study population size of *n* = 1000, evenly distributed in terms of age and sex (age range of 45–69 years). An invitation letter was sent by post, and signing and returning the reply form constituted the provision of informed consent. The response rate was 62 %. The data collection was conducted from October 2003 to June 2004 and constituted a brief health examination at a PHC, collection of morning blood samples in a fasting state, and self-reported measures evaluated by questionnaire. Subjects were instructed not to attend when they were experiencing any acute infections. Exclusion criteria were self-reported severe diseases, such as terminal cancer, severe dementia, and psychiatric disorders. The study sample was representative of the population in terms of educational attainment, immigrant status, and rate of employment. The study was approved by the Regional Ethics Review Board of Linköping University, Linköping, Sweden (02–0324).

#### Medical Conditions and Other Cardiovascular Risk Factors

Medical conditions related to inflammation were covered a priori with a checklist regarding whether the participants had ever been diagnosed by a doctor with any of the following conditions: acute coronary syndrome (ACS), angina pectoris, stroke, chronic obstructive pulmonary disease (COPD), cancer, asthma, allergy, and peptic ulcer. Furthermore, diagnoses of rheumatic disease, fibromyalgia, and herniated disks were captured using the question “Have you ever had any other chronic or long-term disease diagnosed by a medical doctor? (Yes/no/do not know. If yes, please specify).”

Smoking habits were based on self-reports of being a regular smoker (yes, at least one cigarette a day/no) or not. Those who had quit smoking within the last 5 years due to illness were also included in the smoker category. Questions about fruit and vegetables and alcohol consumption were from the validated Food Frequency Questionnaire adopted from the Swedish Mammography Cohort [28]. Physical activity was measured using questions about daily physical activity and planned physical exercise [29]. The two middle categories of the original four were merged into one middle category to minimize ordinal misclassification.

The individuals’ weight and height were obtained during visits to the PHC and used for calculating body mass index (BMI). Systolic and diastolic blood pressures (SBP and DBP) were measured three times in a sitting position after 5 min of rest (Omron M5-1 digital). The mean of the second and third measurements was used. Blood lipids and glucose were analyzed directly after sample collection (ADVIA 1650 and Hemocue glucose system).

#### Psychological Assessments

The LSH study uses validated psychometric instruments that have been previously used in population based studies and that have a plausible predictive value for new coronary events.

Three measures of psychological resources were used in this specific study, namely, coping, self-esteem, and SOC. These were shown in an 8-year follow-up of the LSH study to predict new coronary events, independently of cardiovascular risk factors [12]. Coping captures feelings of confidence and self-reliance with an experience that life to some extent is manageable and was measured with Pearlin and Schooler’s seven-item questionnaire [30]. Self-esteem was measured by the Pearlin’s ten-item adaptation of Rosenberg’s self-esteem [31], which contains both a global dimension of basic self-worth and a comparison with other people’s competences [30]. SOC was measured by the 13-item version of the scale by Antonovsky which assesses to what extent life is regarded as manageable, comprehensive, and meaningful [32].

In addition, Cantril’s measure of global quality of life (ladder of life) was used as a measure of life satisfaction and positive outlook [33].

Three measures of psychological risk factors were used, namely, hopelessness, depressive symptoms, and vital exhaustion. Hopelessness was measured by the Everson’s 2-item scale [7] and depressive symptoms by the 20-item questionnaire from the Center for Epidemiological Studies Depression Scale (CES-D) [34]. Vital exhaustion was measured by the 19-item questionnaire that captures feelings of physical and mental fatigue [35].

#### Biochemical Analyses

Levels of IL-6 were measured in EDTA plasma using Ultrasensitive Bead Kit Technology (Invitrogen Co., Carlsbad, CA, USA) on a Luminex^®^ 100^™^ System (Austin, TX, USA). The lower detection limit was set at 1.68 pg/mL for IL-6. The inter-assay coefficient of variation (CV%) was 7.0 %. The detection rate was 40 %. Due to the low detection rate, IL-6 was also analyzed in a randomly selected subset of the study population (*n* = 380) by a highly sensitive ELISA immunoassay (Quantikine HS, R&D Systems Europe Ltd., Abingdon, UK), with a detection rate of 100 % (detection limit 0.04 pg/mL and CV% 7.8 %).

CRP was measured in plasma by a highly sensitive latex-enhanced turbidimetric immunoassay (Roche Diagnostics GmbH, Vienna, Austria) with a lower detection limit of 0.03 mg/L (CV% 1.6 %) and with a detection rate of 100 %.

Concentrations of MMP-9 were measured in EDTA plasma [36] using human Biotrak ELISA systems (Amersham Biosciences, Uppsala, Sweden). The assay for MMP-9 measures total quantification, that is, biologically free MMP-9, along with inactive pro-MMP-9 and the complex pro-MMP-9/tissue inhibitor of metalloproteinases-1. The lower detection limit was 0.6 ng/mL (CV% 6.8 %) and the detection rate was 100 %. Aliquots of plasma (0.5 mL) were stored at −70 °C for approximately 18 months before laboratory analysis of MMP-9 in a subset of the sample population (*n* = 400) and approximately 40 months before analyses of the remaining MMP-9 samples and the other biomarkers. Analyses by ELISA were run in duplicates.

#### Statistical Analysis

Circulating levels of the biomarkers IL-6, CRP, and MMP-9 were set as the main outcomes. In order to avoid an influence of acute infection, all subjects with CRP levels higher than the clinically accepted cutoff of 10 mg/L were excluded (*n* = 32, mean 20.6 mg/L). Further, all IL-6 levels over >20 pg/mL were excluded (*n* = 17, mean 73 pg/mL), based on a cutoff of 3 SD higher than mean after exclusion of extreme outliers (about hundredfold the mean, >200 pg/mL). Likewise, four values of MMP-9 were more than 3 SD higher than any of the others (cutoff >200 mg/mL and mean 262 ng/mL) and were excluded prior to analyses.

Chi-squared tests and *t* tests were performed to test for group differences. Pearson’s product–moment correlations and linear regressions were used to test associations between variables. A set of linear regression models were run to test the associations between psychological instruments and inflammatory markers. There were three steps as follows: (a) adjustments solely for age and sex; (b) adjustments for age, sex, and medical conditions related to inflammation (ACS, angina pectoris, stroke, COPD, rheumatic disease, asthma, cancer, fibromyalgia, peptic ulcer, and herniated disks); and (c) adjustments for the age, sex, and medical conditions related to inflammation plus cardiovascular risk factors, smoking, alcohol intake, fruit and vegetable intakes, physical activity, BMI, SBP, blood glucose, and HDL. Further, psychosocial resources were adjusted for psychosocial risk factors and vice versa in post hoc analyses. In these analyses, depressive symptoms were included in two ways, as a dichotomous variable using a CES-D cutoff score of 16 or higher (since this has been suggested to discriminate clinically significant depressive symptoms in a general population [34]) or as a continuous score of CES-D.

Owing to a skewness in the distribution of the biomarkers, all of the above models were run with both continuous and log-transformed outcomes.

A two-sided probability value of *p* ≤ 0.05 was considered as statistically significant. Analyses were performed using STATA statistical software, release 11.0 (Stata Corporation, College Station, TX, USA), and IBM SPSS for Windows statistical software, release 21 (IBM Corporation, Armonk, NY, USA).

## Results

Data were available for 944 participants after exclusion of abnormal levels of inflammatory markers. Descriptive characteristics are given in Table 1. Women scored significantly lower than men on coping (*p* = 0.019) and self-esteem (*p* = 0.002) but significantly higher on hopelessness, depression, and vital exhaustion (*p* for all <0.001). In addition, women had significantly higher CRP values (*p* = 0.007) but lower MMP-9 values (*p* < 0.001), while the levels of IL-6 did not differ significantly between women and men (data not shown).

**Table 1:** Descriptive characteristics of the study population

–	Total number	Number (%)	Mean (SD)
Sex	944	–	–
Women	–	473 (50)	–
Age	944	–	–
45–49 years	–	184 (19)	–
50–54 years	–	192 (20)	–
55–60 years	–	195 (21)	–
60–65 years	–	188 (20)	–
65–69 years	–	185 (20)	–
Educational attainment	927	–	–
9 years or less	–	329 (35)	–
10–11 years	–	276 (30)	–
12–13 years	–	128 (14)	–
College/university	–	194 (21)	–
Lifestyle factors			
Smoking (yes)	880	90 (20)	–
Alcohol consumption	923	–	–
Low consumption	–	744 (80)	–
Elevated consumption	–	89 (10)	–
High consumption	–	90 (10)	–
Physical activity	876	–	–
Regularly active	–	167 (19)	–
Occasionally/seldom active	–	670 (76)	–
Inactive	–	39 (4)	–
Fruit and vegetable intakes	926	–	–
Low intake	–	127 (14)	–
Medium intake	–	661 (71)	–
High intake	–	138 (15)	–
Physical factors			
BMI (kg/m^2^)	935	–	26.8 (4.2)
SBP (mm Hg)	931	–	133 (20.2)
DBP (mm Hg)	931	–	84 (11.6)
LDL cholesterol (mmol/L)	921	–	3.4 (0.88)
HDL cholesterol (mmol/L)	937	–	1.5 (0.36)
Plasma glucose (mmol/L)	932	–	5.4 (1.2)
Inflammatory medical conditions^a^	944	184 (19)	–
Biomarkers			
IL-6 (pg/mL) Luminex	944	–	2.0 (2.7)
IL-6 (pg/mL) ELISA	377	–	2.2 (1.7)
CRP (mg/L)	928	–	1.7 (2.0)
MMP-9 (ng/mL)	903	–	35.2 (23.1)

^a^Defined a priori as participants diagnosed with ACS, angina pectoris, stroke, COPD, cancer, asthma, allergy, peptic ulcer, rheumatic disease, fibromyalgia, and/or herniated disk

Table 2 shows the associations of inflammatory biomarkers with cardiovascular risk factors. IL-6 values from ELISA (*n* = 377) were chosen instead of IL-6 for Luminex (*n* = 944) as the former had a detection rate of 100 %. Abundant significant associations were shown between cardiovascular risk factors and levels of IL-6, CRP, and MMP-9.

**Table 2:** Cardiovascular risk factors in relation to inflammatory biomarkers

	IL-6 (*n* = 344)		CRP (*n* = 928)		MMP-9 (*n* = 903)	
	*β*	*p*	*β*	*p*	*β*	*p*
Sex (women vs. men)	−0.36	0.040	0.35	0.002	−7.38	<0.001
Age (5-year categories)	0.28	<0.001	0.14	0.007	−0.56	0.294
Lifestyle factors						
Smoking (yes/no)	0.50	0.031	0.55	0.002	12.87	<0.001
Alcohol intake (three categories)	0.32	0.023	−0.13	0.059	1.82	0.025
Fruit/vegetable intake (three categories)	0.10	0.544	−0.01	0.935	−3.90	0.006
Physical activity (three categories)	−0.43	0.029	−0.54	<0.001	−8.24	<0.001
Physical factors						
BMI (three categories)	0.64	<0.001	0.89	<0.001	3.67	<0.001
SBP (SD 20 mm Hg)	0.25	0.005	0.25	<0.001	2.70	0.001
DBP (SD 11 mm Hg)	0.21	0.010	0.27	<0.001	2.19	0.004
LDL cholesterol (SD 0.87 mmol/L)	−0.17	0.070	−0.03	0.615	1.0	0.185
HDL cholesterol (SD 0.36 mmol/L)	−0.37	<0.001	−0.46	<0.001	−3.4	<0.001
Glucose (SD 1.2 mmol/L)	0.25	0.006	0.27	<0.001	0.56	0.461
Inflammatory medical condition^a^	0.53	0.022	0.36	0.029	4.70	0.013
Biomarkers						
IL-6 (pg/mL)	–	n.a.	0.89	<0.001	4.95	0.001
CRP (mg/L)	0.61	<0.001	–	n.a.	7.74	<0.001
MMP-9 (ng/mL)	0.51	<0.001	0.51	<0.001	–	n.a.

Regressions adjusted for age and sex. Βeta coefficient expressed as an increase of circulating levels per dichotomy, category, or SD increment

^a^Defined a priori as participants diagnosed with ACS, angina pectoris, stroke, COPD, cancer, asthma, allergy, peptic ulcer, rheumatic disease, fibromyalgia, and herniated disks at baseline

Table 3 shows the associations of cardiovascular risk factors with psychological factors. Regarding self-esteem, there were non-significant associations to all investigated factors except sex, fruit intake, BMI, and medical conditions (*p* < 0.01). BMI was associated with all other psychological factors (*p* < 0.05); the associations were negative for resources and positive for risk factors. Smoking was associated with all psychological risk factors and one psychological resource, the ladder of life (*p* < 0.05). Physical activity showed a similar pattern of associations to all psychological factors (*p* < 0.05), except coping and self-esteem. Fruit intake was associated with all resources except the ladder of life and one risk factor, hopelessness (*p* < 0.05). Alcohol intake was associated with one resource, SOC, and one risk factor, vital exhaustion (*p* < 0.05). Neither blood glucose nor blood pressure or blood lipids were associated with any of the psychological factors, with the exception of the association of HDL with hopelessness (*p* < 0.05).

**Table 3:** Cardiovascular risk factors in relation to psychological resources and risk factors

	Coping (*n* = 890 to 948), range 7–28		Self-esteem (*n* = 890 to 947), range 0–40		SOC (*n* = 904 to 962), range 13–91		Ladder of life (*n* = 840 to 895), range 0–10		Hopelessness (*n* = 904 to 963), range 0–8		Vital exhaustion (*n* = 907 to 965), range 17–57		CES-D (*n* = 884 to 963), range 0–60	
	*β*	*p*	*β*	*p*	*β*	*p*	*β*	*p*	*β*	*p*	*β*	*p*	*β*	*p*
Sex (women vs. men)	−0.76	0.001	−0.99	0.001	−0.66	0.318	−0.13	0.233	0.45	<0.001	2.99	<0.001	2.23	<0.001
Age (5-year categories)	−0.06	0.397	−0.04	0.680	1.10	<0.001	0.15	<0.001	0.21	<0.001	−0.55	0.001	−0.39	0.029
Lifestyle factors														
Smoking (yes/no)	−0.46	0.084	−0.53	0.155	−1.32	0.100	−0.28	0.040	0.38	0.013	2.11	<0.001	2.92	<0.001
Alcohol intake (three categories)	−0.20	0.275	−0.28	0.271	−1.81	0.001	−0.13	0.141	0.03	0.738	0.85	0.032	0.65	0.117
Fruit/vegetable intake (three categories)	0.53	0.013	1.03	<0.001	1.36	0.032	0.13	0.200	−0.33	0.005	−0.36	0.427	−0.83	0.088
Physical activity (three categories)	0.45	0.068	0.32	0.353	1.81	0.018	0.25	0.038	−0.52	<0.001	−1.60	0.003	−1.39	0.012
Physical factors														
BMI (three categories)	−0.31	0.037	−0.32	0.099	−1.08	0.018	−0.20	0.008	0.32	<0.001	1.21	<0.001	0.86	0.014
SBP (SD 20 mm Hg)	−0.12	0.282	−0.14	0.149	0.03	0.929	0.02	0.755	0.12	0.076	−0.08	0.725	−0.04	0.877
DBP (SD 11 mm Hg)	−0.15	0.181	−0.25	0.122	−0.09	0.769	−0.02	0.676	0.13	0.052	0.06	0.980	0.07	0.792
LDL cholesterol (SD 0.87 mmol/L)	−0.09	0.423	−0.10	0.499	−0.09	0.794	−0.04	0.411	0.00	0.962	−0.10	0.672	0.11	0.665
HDL cholesterol (SD 0.36 mmol/L)	0.11	0.378	0.05	0.766	0.30	0.394	0.07	0.236	−0.14	0.038	−0.20	0.434	−0.12	0.656
Glucose (SD 1.2 mmol/L)	0.20	0.071	0.28	0.068	−0.05	0.872	−0.06	0.295	0.05	0.473	0.34	0.150	0.03	0.915
Inflammatory medical condition^a^	−0.93	0.001	−1.19	0.002	−3.56	<0.001	−0.62	<0.001	0.66	<0.001	3.89	<0.001	2.58	<0.001

Regressions adjusted for age and sex. Βeta coefficient expressed as an increase of score in psychological instrument per dichotomy, category, or SD increment

*SOC* sense of coherence, *CES-D* Center of Epidemiology Studies Depression Scale

^a^Defined a priori as participants diagnosed with ACS, angina pectoris, stroke, COPD, cancer, asthma, allergy, peptic ulcer, rheumatic disease, fibromyalgia, and herniated disks at baseline

All psychological measures are significantly correlated with each other. The lowest correlation is found between hopelessness and ladder of life (*r* = −0.36). The highest correlation is found between CES-D and vital exhaustion (*r* = 0.76).

Table 4 shows the results from a set of regression models on the inflammatory biomarkers IL-6 (using ELISA analyses), log-CRP and MMP-9 with psychological measures as independent variables. All analyses were carried out on both continuous and log-transformed data of the biomarkers. The log transformation did not alter the associations for the biomarkers, except for CRP. Continuous CRP was significantly associated with one of the psychological measures (ladder of life) but to six measures when log-transformed CRP was used. The analyses on IL-6 by the Luminex method corroborate the findings on IL-6 using ELISA, despite a low detection rate. In the full regression models, the same significant associations were noted to psychological measures shown in Table 4, except for CES-D (*p* = 0.138). However, all regression coefficients were lower when using Luminex instead of ELISA measures (ranging from −0.19 to 0.22 pg/mL per SD increment).

**Table 4:** Psychological factors in relation to IL-6, CRP, and MMP-9 after adjustments

IL-6 (*n* = 288 to 329)	Model a		Model b		Model c	
	*β*	*p*	*β*	*p*	*β*	*p*
Coping (SD 3.4)	−0.37	<0.001	−0.35	<0.001	−0.29	0.003
Self-esteem (SD 4.8)	−0.34	0.001	−0.32	0.001	−0.28	0.004
SOC (SD 10.4)	−0.23	0.024	−0.21	0.039	−0.15	0.136
Ladder of life (SD 1.7)	−0.50	<0.001	−0.49	<0.001	−0.38	<0.001
Hopelessness (SD 2.0)	0.27	0.014	0.24	0.027	0.19	0.071
Depression CES-D (SD 7.8)	0.41	<0.001	0.39	<0.001	0.27	0.010
Vital exhaustion (SD 7.6)	0.39	<0.001	0.36	0.001	0.26	0.017
Log-CRP (*n* = 703–803)						
Coping (SD 3.4)	−0.08	0.078	−0.08	0.098	−0.05	0.291
Self-esteem (SD 4.8)	−0.11	0.021	−0.11	0.016	−0.09	0.037
SOC (SD 10.4)	−0.09	0.047	−0.10	0.043	−0.06	0.194
Ladder of life (SD 1.7)	−0.12	0.008	−0.15	0.004	−0.09	0.036
Hopelessness (SD 2.0)	0.12	0.014	0.10	0.042	0.04	0.434
Depression CES-D (SD 7.8)	0.12	0.012	0.10	0.046	0.06	0.179
Vital exhaustion (SD 7.6)	0.11	0.021	0.09	0.069	0.04	0.444
MMP-9 (*n* = 688–784)						
Coping (SD 3.4)	−1.32	0.094	−1.45	0.085	−0.92	0.253
Self-esteem (SD 4.8)	−1.74	0.030	−2.05	0.016	−1.67	0.040
SOC (SD 10.4)	−2.17	0.006	−2.38	0.005	−1.84	0.026
Ladder of life (SD 1.7)	−1.67	0.038	−1.54	0.075	−1.05	0.204
Hopelessness (SD 2.0)	1.02	0.195	0.94	0.280	0.18	0.833
Depression CES-D (SD 7.8)	1.84	0.022	1.91	0.026	1.10	0.185
Vital exhaustion (SD 7.6)	1.29	0.107	1.18	0.172	0.52	0.538

Βeta coefficient expressed as increase of circulating levels of IL-6 (pg/mL), CRP (mg/L), and MMP-9 (ng/mL) per SD increment. Coefficient for CRP is log-transformed in regression and then back-transformed in table

*Model a* adjusted for age and sex; *model b* adjusted for age, sex, and medical conditions (angina pectoris, ACS, stroke, COPD, rheumatic disease, asthma, cancer, fibromyalgia, peptic ulcer, and herniated disks); and *model c* adjusted for age, sex, and medical conditions (smoking, alcohol intake, fruit and vegetable intakes, physical activity, body mass index, blood pressure, blood lipids, and blood glucose)

The regression coefficients are relatively small, but the associations are found in expected directions. After adjustment for age and sex, significant associations were found for all psychological factors with IL-6, for all but coping with log-CRP and for all but three (coping, hopelessness, and vital exhaustion) for MMP-9. Controlling for presence of somatic disease in the second step of the regression models displayed small effects with loss of significant relationships between the ladder of life and MMP and vital exhaustion for CRP. After full adjustments also for medical conditions and cardiovascular risk factors, self-esteem was independently associated with all three biomarkers (*p* < 0.05). Ladder of life was associated with IL-6 and log-CRP (*p* < 0.05); coping, vital exhaustion, and depressive symptoms with IL-6 (*p* < 0.05); and SOC with MMP-9 (*p* < 0.05).

In post hoc analyses, we used the model “c” presented in Table 4 but adjusted psychological resources for psychological risk factors and vice versa. Overall, hopelessness, depressive symptoms (either as continuous or dichotomous variable), and vital exhaustion had low effect on the association between psychosocial resources and inflammatory markers. There were only two specific models in which a psychological risk factor was significantly associated with the inflammatory marker at hand when entered together with a psychological resource. Those associations were found between IL-6 and depressive symptoms (dichotomous) entered either with coping or with ladder of life (*p* for CES-D = 0.042 and 0.047, respectively).

For resources and ladder of life, on the other hand, most associations with inflammatory markers remained after adjustment for psychosocial risk factors. Coping remained associated with IL-6 regardless of adjustment for hopelessness, vital exhaustion, or depressive symptoms (continuous or dichotomous). Self-esteem remained significantly associated with IL-6, CRP, and MMP-9 also after adjustment for hopelessness, vital exhaustion, or depression using dichotomy scale and with marginal significance for IL-6 (*p* = 0.06) and CRP (*p* = 0.061) after control for depression using continuous scale.

Sense of coherence remained significantly associated with MMP-9 after adjustment for vital exhaustion or CES-D (dichotomous) but marginally when adjusted for hopelessness (*p* = 0.052) and CES-D (continuous, *p* = 0.100).

Ladder of life remained significantly associated with IL-6 regardless of adjustment for any of the psychosocial risk factors but was not with associated with CRP after adjustment for any of those.

## Discussion

This study investigated associations between psychological and inflammatory predictors of CHD in a community-based sample. Significant associations of psychological resources and risk factors with IL-6, CRP, and MMP-9 were shown. The associations are consistently found in expected directions, with psychological resources associated with lower levels of inflammatory markers and psychological risk factors associated with higher levels of inflammatory markers. Interestingly, psychological resources associated with other cardiovascular risk factors to a lower degree than is the case for psychological risk factors. As a consequence, adjustments for cardiovascular risk factors have a higher impact on the association between psychological risk factors and inflammatory markers.

One important notion is that several of the significant associations between psychological resources remain after adjustment for psychological risk factors. Thus, the measures may appear similar but do capture different psychological constructs and are therefore not redundant measures.

Of note, self-esteem displayed independent associations to all the three inflammatory markers. This is interesting in view of our previous findings, highlighting self-esteem as an independent predictor of CHD events, also after control for depression or hopelessness [12]. Hence, self-esteem may be worth considering in the search of future health predictors and for planning of preventive actions against CHD events. The self-esteem scale aims to capture the positivity of one’s attitudes toward oneself and self-worth [30].

The findings of independent significant associations of ladder of life with IL-6 and CRP are in line with prior studies on positive affect and life satisfaction in women [37] as well as on optimism in older men [38]. To our knowledge, this instrument has not been used before in psychoimmunological investigations. It is suggested as a relevant measure when scrutinizing the interplay between psychological dimensions and immunologic markers.

The association between MMP-9 and SOC supports earlier published results from a subset of this study [23]. MMP-9 is a rather new biomarker in the field of psychoneuroimmunology, which makes this finding interesting but hard to evaluate until further research has accumulated on this topic.

In agreement with several earlier studies [18–21], IL-6 was the biological marker most frequently associated with psychological factors, thus confirming that IL-6 is a robust marker in the field of psychoneuroimmunology. Contradicting the Multi-Ethnic Study of Atherosclerosis (MESA) and the Whitehall II studies [37, 39], significant associations of IL-6 mostly remained after adjustments for cardiovascular risk factors, even in a separate post hoc analysis simulating the MESA study model (not shown) [39].

As compared with IL-6, a larger proportion of associations to CRP and MMP-9 became non-significant after adjustments for other cardiovascular risk factors. This suggests that the relations of psychological measures to MMP-9 and CRP depend on cardiovascular risk factors to a higher extent than is the case for IL-6 thereby suggesting that biomarkers related to inflammation should not be used interchangeably but rather be treated as markers of different immunological functions.

As for psychological risk factors, their influences on inflammatory markers seem to be associated to a larger degree with cardiovascular risk factors. Of note, neither psychological risk factors nor resources showed significant associations with levels of glucose, blood pressure, or blood lipids. Therefore, it is plausible that the associations of hopelessness with IL-6 and CRP and of depression with CRP and MMP-9, all of which vanished after adjustment for physical and lifestyle factors, are mainly reliant on lifestyle factors. The lifestyle factors, smoking and physical activity and subsequently BMI, may be of particular importance for these associations as linked with all the involved parameters.

Interestingly, the presence of medical conditions only marginally influenced the associations between inflammatory markers and psychological factors in line with previous research [40]. Only the associations of vital exhaustion with CRP as well as of the ladder of life with MMP-9 seem to be confounded by inflammatory medical conditions. This seems reasonable as vital exhaustion may be a part of somatic disorder which in turn also may moderate optimism, approximated by the ladder of life.

### Limitations and Strengths

The high proportion of measured samples with cytokine levels under the detection level is of concern. However, it was possible to validate the findings on IL-6 levels by using a high-sensitivity ELISA approach on a subset of the study population. The two different laboratory techniques generated consistent results in regression models, although the ELISA method provided slightly larger effect sizes. This was probably due to the ability of the ELISA method to quantify also lower levels of IL-6.

The choice to have inflammatory markers as a dependent outcome may be a matter of some controversies as the direction could be the other way around, namely, that the cytokines contribute to psychological disposition [18, 41]. As our primary interest was to study through which pathways psychological factors exert their effects on CHD, we chose inflammatory markers as a dependent outcome in this study.

The number of analyses performed may increase the risk of alpha errors. We have chosen not to adjust for mass significance (for example, Bonferroni correction) as it might over-adjust significance level due to high correlations between the psychological measures. Due to the collinearity, the risk of beta-errors is high too, in particular, in the regression models where both resources and risk factors were entered at the same time. Thus, these regressions should be interpreted with caution.

There are a number of strengths in this study of a large-scale well-characterized community-based sample. One particular benefit is the broad range of psychological constructs of both resources and risk factors. Despite the relatively small number of participants in comparison to other large-scale community-based studies, we were able to demonstrate significant findings in expected directions for several psychological measures, even after broad adjustment for confounders.

## Conclusion

Numerous significant associations of psychological resources and risk factors with IL-6, CRP, and MMP-9 were found in a community-based sample. The associations of psychological resources were mostly independent, while the psychological risk factors seemed preferentially dependent on lifestyle factors as smoking, physical activity, and BMI. This suggests that the psychological resources’ (in particular self-esteem) protective effects on CHD are linked to inflammatory markers.

## Abbreviations

ACS: Acute coronary syndrome
BMI: Body mass index
CES-D: Center of Epidemiological Studies Depression Scale
COPD: Chronic obstructive pulmonary disorder
CRP: C-reactive protein
DBP: Diastolic blood pressure
ELISA: Enzyme-linked immunoassay
HDL: High-density-lipoprotein cholesterol
IL: Interleukin
LDL: Low-density-lipoprotein cholesterol
LSH: Life Conditions, Stress, and Health study
MESA: Multi-Ethnic Study of Atherosclerosis
MMP: Matrix metalloproteinase
PHC: Primary healthcare center
SOC: Sense of coherence
SBP: Systolic blood pressure
